# The Comparison of Physicochemical Parameters, Antioxidant Activity and Proteins for the Raw Local Polish Honeys and Imported Honey Blends

**DOI:** 10.3390/molecules26092423

**Published:** 2021-04-21

**Authors:** Michał Miłek, Aleksandra Bocian, Ewelina Kleczyńska, Patrycja Sowa, Małgorzata Dżugan

**Affiliations:** 1 Department of Chemistry and Food Toxicology, Institute of Food Technology and Nutrition, University of Rzeszów, Ćwiklińskiej 1a, 35-601 Rzeszów, Poland; ewelina1213@poczta.onet.pl (E.K.); mdzugan@ur.edu.pl (M.D.); 2 Department of Biotechnology and Bioinformatics, Faculty of Chemistry, Rzeszów University of Technology, Powstańców Warszawy 6, 35-959 Rzeszów, Poland; bocian@prz.edu.pl; 3 Department of Bioenergetics Food Analysis and Microbiology, Institute of Food Technology and Nutrition, University of Rzeszów, Ćwiklinskiej 2D, 35-601 Rzeszów, Poland; psowa@ur.edu.pl

**Keywords:** honey, quality standards, protein, amylase, acid phosphatase, native PAGE

## Abstract

Many imported honeys distributed on the Polish market compete with local products mainly by lower price, which can correspond to lower quality and widespread adulteration. The aim of the study was to compare honey samples (11 imported honey blends and 5 local honeys) based on their antioxidant activity (measured by DPPH, FRAP, and total phenolic content), protein profile obtained by native PAGE, soluble protein content, diastase, and acid phosphatase activities identified by zymography. These indicators were correlated with standard quality parameters (water, HMF, pH, free acidity, and electrical conductivity). It was found that raw local Polish honeys show higher antioxidant and enzymatic activity, as well as being more abundant in soluble protein. With the use of principal component analysis (PCA) and stepwise linear discriminant analysis (LDA) protein content and diastase number were found to be significant (*p* < 0.05) among all tested parameters to differentiate imported honey from raw local honeys.

## 1. Introduction

Honey is a product with a diverse chemical composition, which depends mainly on the type and species of plant from which it originates. Poland is distinguished by particularly large beekeeping traditions and Polish honeys invariably have a good reputation in foreign markets. Lately, a lot of low-price imported honeys available on the Polish and EU market have been competing with local products [1]. When the honey is a blend of honeys harvested from more than one country, placing exact information concerning the country of origin on the label is not required [2]. Imported honey is usually labeled as: “blend of EU honeys”, “blend of non-EU honeys”, or “blend of EU and non-EU honeys”. Such honeys can be of poor quality due to processing to extend their shelf life. Research conducted by Dżugan et al. (2018) showed that imported honeys had an increased content of HMF (5-hydroxymethylfurfural) and reduced diastase number, electrical conductivity, and total acidity as compared to raw local honeys [3]. Imported honeys are frequently thermally processed to kill certain types of bacteria or yeast responsible for fermentation and prevent crystallization during storage. However, such processing also removes the natural flavors and reduces antibacterial properties, nutrients, and antioxidants content. Contrarily, local raw honey is seen as a high-quality and less allergenic product due to its pollen origin from the immediate locations. Moreover, local raw honey containing pollen from the surrounding area is known to immunize allergy sufferers, especially children [4,5].

Until now the comparison between local and imported blends has been rarely performed. Simultaneously, in numerous studies multivariate statistical analysis has been applied for differentiating honey samples based on physicochemical properties and the content of biologically active compounds [6,7,8]. Such classification is performed mainly based on the biological origin of the samples, less often the geographical one. These analyses can be also used to detect adulteration in honey. Despite a large spectrum of indicators used in the honey analysis, starting with simple physicochemical parameters evaluation, pollen analysis, sugar profiles by HPLC analysis, up to stable carbon isotope ratio analysis (SCIRA) based on the calculation of the 13C/12C isotopes ratio [9,10,11], there is still an urgent need to implement an effective method to differentiate the quality of honey samples. Thus, the big challenge is applying chemometric analyses in the area of the differentiation of raw local honeys and available on the market blends.

Raw honeys are generally more abundant in proteins which, due to their thermola-bility, seem to be a sensitive but not frequently used marker of honey quality. Honey proteins are mainly of bee origin, and only part of them come from nectar [12]. The protein content is variety-dependent (0.2–0.4 mg/100 g for blossom and 0.4–0.7 mg/100 g for honeydew honey) [13] and thermal processing affects protein level negatively. These molecules are found in honey in small amounts, but they are partly responsible for the healing properties of honey. Although natural honeys contain a small amount of enzymes, including diastase, invertase, glucose oxidase, acid phosphatase, catalase, and β-glucosidase, they are very important in creating honey bioactivity [14]. Only diastase activity is included in honey standards [15]. By using electrophoresis SDS-PAGE, protein fractions can be obtained, and the number of proteins and polypeptides, as well as their molecular weight, can be determined. However, electrophoretic techniques are rarely used in honey analysis, although they can be a good tool for assessing the protein profile and even zymographic detection of individual enzymes [16]. Confirmation of the suitability of this technique would provide a promising tool to evaluate the quality of honey.

The aim of this study was to compare the quality of raw local honeys and imported honey blends available on the Polish market based on antioxidant potential, amylase and phosphatase activities, and protein profiling by native PAGE. Multivariate analysis (PCA and LDA) applied to the obtained results allowed us to verify tested samples according to their origin.

## 2. Results

### 2.1. Standard Quality Parameters

In order to assess the quality of tested imported honey in accordance with legal regulations, their physicochemical parameters were determined. These data were compared to reference honey samples originating from an ecological apiary of the Podkarpackie region (Table 1).

Tested honeys predominantly fulfilled applicable legal standards regarding their physicochemical properties. The water content in most of the tested honeys was within the legal limits which was set to be below 20%, except for heather honey (maximum of 23%) [2], but 25% of samples (including controls) slightly exceeded the set limit. However, the water content was variety-dependent; the lowest was determined in acacia honey (17.71% on average) as well as two samples of honeydew honey (17.55%), and the highest in buckwheat and linden (average values 20 and 19.94%, respectively). Increased water content may be caused by adverse weather conditions prevailing when honey was produced by bees or immaturity resulting from early acquisition from the hive [13]. The results of the analysis of free acids contained in the tested honeys prove that these honeys were mostly within the norm and were comparable to our earlier findings [3,17,18]. The acidity of honey depends on the type of raw material, the season in which it was obtained, and the degree of its maturity [13]. Organic acids contained in honey lower its pH, which prevents the growth of microorganisms and extends the product′s shelf life. As honey conductivity should be within the range of 0.2 to 0.8 mS/cm for nectar and above 0.8 mS/cm for honeydew honey [2], all tested honeys fell within these parameters. This parameter allows the distinguishing between nectar and honeydew honeys easily. The analysis by Tomczyk et al. [18] of the physicochemical properties of selected nectar honey varieties from the Podkarpackie region showed the conductivity of nectar honey ranged from 0.23 (for rape) to 0.82 mS/cm (for forest honey) which is comparable to the values obtained in the present study.

The HMF (5-hydroxymethylfurfural) content in honey must not exceed 40 mg/kg [2]. Among the samples tested, only two imported multifloral honeys showed HMF content higher than permissible standards. However, in most cases, raw honeys from the Podkarpackie region contained less HMF than their imported counterparts, except for the lower values for single samples of acacia, buckwheat, and honeydew honey. As an HMF increase can result from long storage in inappropriate conditions, adulteration with corn syrup, or prolonged heating, it is an important parameter used to control honey overheating. Such processing may cause a decrease in the nutritional value by degradation of thermolabile vitamins and bio-nutrients, and also contribute to a decrease in diastase activity [19]. It is in agreement with the findings of Sanz et al. for honeys obtained directly from beekeepers, which contained approximately five times lower HMF than honeys purchased in a supermarket [20]. The increased level of HMF content in the case of some imported honeys may result from their long storage or from the use of heating the honey in the production of blends.

### 2.2. Antioxidant Properties

Antioxidant properties of honey are not specified in legal regulations; however, they have been proposed as a useful indicator in the authentication of honey botanical origin [7]. On the other hand, antioxidant activity can serve simply as an indicator of the biological activity of honey measured in vitro. A higher antioxidant capacity determines better antimicrobial and anti-inflammatory activity of honey [21,22]. The data regarding the antioxidant properties of all analyzed honeys are shown in Table 2. The total phenolic content was significantly correlated with the results of FRAP and DPPH analysis (Pearson coefficient 0.952 and 0.558, respectively). The different strength of the correlation results from the different mechanisms of the two methods used, which differ in sensitivity against the hydrophilic and hydrophobic antioxidants fraction. The study showed a diverse content of phenolic compounds depending on the honey type. Dark honeys (buckwheat and honeydew) showed a higher content of these compounds. This is a well-known feature of honey: the darker the honey, the richer in polyphenols, which was previously presented by several authors [22,23,24]. Comparing ecological Podkarpackie honeys with imported honey blends within the same variety, even the several times higher polyphenol content and antioxidant activity of Polish nectar honeys (*p* < 0.05) measured by FRAP method can be noticed. For honeydew honeys, smaller differences were observed. Smaller differences for DPPH assay results were observed, which follows from the manner of the results’ expression, as a percent of radical inhibition for direct comparison of the same honey dilution, without calculating it to the honey mass unit. Based on the obtained results of antioxidant potential, health benefits can be expected from consuming local honeys of high quality. Special pro-health properties of honey with a high content of antioxidant compounds were previously proved in the example of buckwheat honey, which showed a protective effect against oxidative stress during an in vitro study using a yeast biological model [22].

### 2.3. Protein Content and Enzyme Activities

Table 2 summarizes also the total protein content and activity of selected enzymes (amylase and acid phosphatase) in tested honeys.

Based on the obtained results, it was found that the protein content strongly depends on the honey variety. The largest amounts of protein were determined in buckwheat and honeydew honeys, which belong to the dark honeys. Acacia honey contained the lowest amount of protein. Comparing ecological honeys with imported blends regardless of the variety, a lower protein content was determined, excluding honeydew honey. Statistically significant differences occurred for honeydew, multi-flower, buckwheat, linden, and acacia honeys. However, the largest difference in protein content between the control and other samples was found in buckwheat honey. Based on the obtained data, it can be assumed that the amount of protein in honey strongly depends on its botanical origin. Cimpoiu et al. presented a similar opinion; analyzing numerous samples of honey they noticed a significant relationship between the amount of protein and the variety of honey [25]. The total protein content was also determined using the Bradford method by Flanjak et al., who demonstrated that the protein content in honey was in the ranges: 21–43 mg/100 g of honey (acacia) and 30–95 mg/100 g of honey (honeydew) [26] which show a similar order of magnitude to the data presented in this study. Especially in the case of buckwheat honey, enhanced protein content was observed. A recognized indicator of honey quality, included in Polish and international legal requirements for honey, is diastase (α-amylase), the enzyme responsible for the hydrolysis of complex sugars. Natural honey does not contain complex sugars, and the function of this enzyme in honey is not fully known [27]. However, the strong presence of amylase was confirmed in tested raw Polish honeys regardless of the variety [17]. The found values of diastase numbers were very diverse, ranging from 2.7 for acacia and multifloral honey up to 66 for buckwheat. Similarly, in the presented study, the highest enzymatic activity of diastase was observed for all tested buckwheat honeys, control honeydew honey, multifloral, and linden honey. Comparing raw local honeys to imported blends, a much lower diastase number was determined in imported honey samples, excluding acacia honey. This phenomenon could be a result of honey thermal processing. Flanjak et al. investigated the enzymatic activity of amylase, comparing the catalytic capacity of amylase in various types of honey [26], and found the high amylolytic activity of honeydew honeys (DN from approx. 12–37) and low activity of acacia honeys (DN 7.5–14). In turn, Bonta et al. found for acacia honey that the values of the diastase number were below the limit specified in the regulations for 60% of tested samples (from 2.6 to 6) [28].

Honey acid phosphatase activity is related to the fermentation processes of honey. This enzyme originates mainly from nectar and pollen and can be used as a parameter for honey characterization [29]. Buckwheat and honeydew honeys possessed the highest enzymatic activity of acid phosphatase, while the smallest activity was found in linden and acacia honeys. It is worth noting that for most honeys obtained from the ecological apiary, a higher value of enzyme activity was determined than for their imported counterparts. Comparable results were obtained by Flanjak et al. for honeys of different varieties [26]. The authors indicate a wide discrepancy in the results of enzyme activity, but also draw attention to the clearly greater enzymatic activity of acid phosphatase in honeydew honeys than in acacia honey, which is in agreement with the results. Similar conclusions were obtained in the further studies of Dżugan and Wesołowska, where the highest acid phosphatase activity was showed in buckwheat, following by honeydew, linden, and multiflorous honey [30].

### 2.4. Protein Profile by PAGE

**Figure 1:**
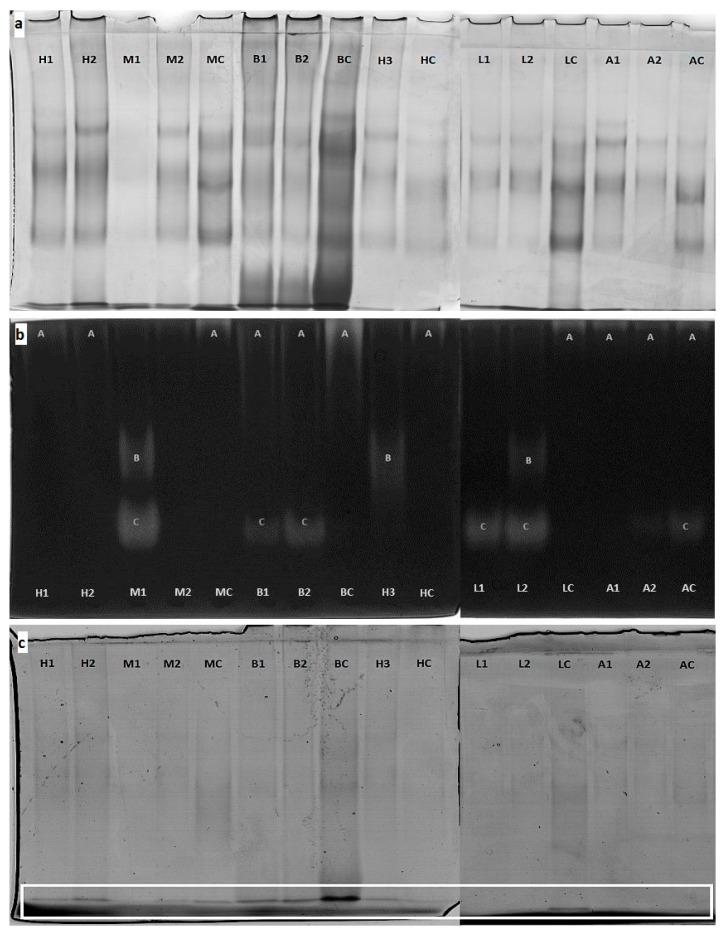
Gels from native PAGE electrophoresis (**a**) for total protein stained with Coomassie Brilliant Blue, (**b**) for amylase activity, and (**c**) for acid phosphatase activity. A,B,C—amylase fraction, the white box marks the location of the acid phosphatase band.

Native electrophoresis gels were stained for protein profile using a colloidal Coomassie Brilliant Blue dye (Figure 1a). It was found that the tested honeys strongly differed in protein profiles, which were manifested in the number of bands and/or their intensity. It was especially visible for buckwheat (especially local BC) and honeydew honeys. The lowest protein content was observed in linden and acacia honeys. It was also noted that organic honey had definitely more protein than imported honey samples of the same variety. The electrophoregrams clearly show that the total number of bands for individual samples strongly varied. The smallest number of bands (3–4) was observed for multiflower honeys and acacia honeys, and the greatest (approx. 6–7) for buckwheat and honeydew honeys. Moreover, organic honey exhibited a richer protein profile than imported ones, excluding honeydew honey. In all samples of Polish honey, the bands visible on the gel are clearer and stronger than in imported samples (Figure 1a). The results obtained indicate that despite not using a marker during native electrophoresis, which is because many different factors affect the speed of protein migration (mass, shape, and charge), it is still an extremely useful technique for screening. Based on electrophoretic separation, it is possible to observe differences in both the amount of protein in samples and the protein profiles of individual honey.

### 2.5. Native Enzymes Detection by PAGE

Amylase activity was detected by native electrophoresis on gels with the addition of starch which were stained with Lugol′s solution (Figure 1b). After staining the gels, bright spots on the gel formed in places where the starch present in the polyacrylamide gel had been digested by the active amylase. This type of electrophoresis is called zymography. Based on the image, it can be assumed that three amylase isoforms (A, B, and C) which differed in electrophoretic migration rates and molecular weights resulted in different positions on the gel. For local honeys, isoform A was specific, excluding acacia honey where isoform C also occurred. Meanwhile, for imported honeys, the isoforms B and C were detected for three and five samples, respectively. Based on the intensity of the bright band, which depends on the activity of enzyme protein, it can be confirmed that Polish buckwheat honey contained the highest amount of active amylase protein, followed by honeydew and linden honeys. The different forms of amylase detected in some imported honeys indicated different origins of the enzyme. The amylase in honey is considered to be mainly of bee origin and is secreted by the salivary and hypopharyngeal glands [31]. However, the presence of proteins with amylolytic activity derived from plants or microorganisms cannot be ruled out [32]. Gels stained for acid phosphatase activity are shown in Figure 1c. In all honey samples, the band corresponding to acid phosphatase was detected in the same place at the bottom of the gel. It may indicate the common source of an acid phosphatase present in honey, and confirmed the idea that this enzyme originated from the honey bee digestive tract [33]. Depending on the honey variety, the color of the bands was more or less intense, which was related to the content of the active enzyme in the samples. Buckwheat honeys were characterized by the highest enzyme activity, whereas lime and acacia honeys were less abundant in acid phosphatase. The ecological Polish honeys (marked with the symbol C) exhibited higher enzyme activity (*p* < 0.05) compared to imported samples of the same botanical origin.

The native electrophoresis is rarely used in the study of honey but allows under non-denaturing conditions to separate isoforms of native proteins with preserved enzymatic activity. Borutinskaite et al. [34] analyzed the enzymatic activity of catalase and glucose oxidase in buckwheat honey using the native PAGE technique and performed an electrophoretic separation of proteins found in buckwheat honey. They showed that buckwheat honey is rich in proteins, as evidenced by a large number of intensely colored protein fractions, and also detected the activity of selected enzymes (catalase and glucose oxidase) on the gel. The use of electrophoresis in denaturing conditions (SDS-PAGE) is more frequently used to verify the honey variety as well as its geographical origin [12,35,36,37]. Some attempts to analyze honey proteins by 2D electrophoresis techniques are also known [16,38]. The authors confirmed the differences in the honeydew and nectar honey proteomes, and they also selected a set of proteins useful for differentiating honey varieties. Based on the PAGE gels presented in this study, it was indicated that native electrophoresis can be a useful tool for the differentiation of local organic honeys and imported blends.

### 2.6. Multivariate Statistical Analysis of Obtained Results

**Figure 2:**
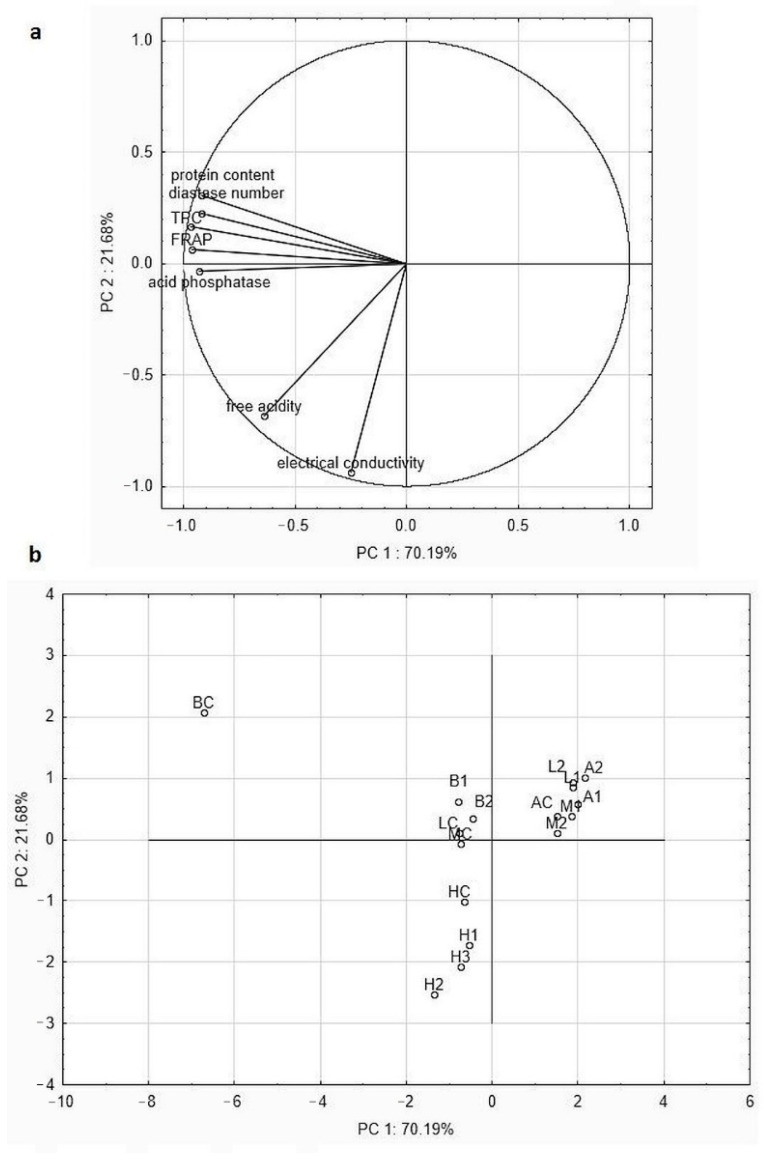
Statistical analysis results: (**a**) projection of chosen variables as a function of the PC1 vs. PC2, (**b**) PCA score plot of imported honey samples available on the Polish market and their ecological local counterparts.

Based on the determined parameters the chemometrics analysis was used to separate imported honey blends from raw honey produced by local beekeepers. MANOVA analysis was applied to determine which variables were statistically dependent (*p* < 0.05) in terms of the botanical or geographical origin of honey samples. The moisture and HMF content, as well as DPPH, were significant only in terms of botanical origin. Other variables were statistically significant in both cases, excluding pH value. None of the variables were statistically significant only due to the geographical origin. For the multivariate analysis, only the seven significant variables (free acidity, electrical conductivity, protein content, diastase number, acid phosphatase activity, total phenolic content, and antioxidant activity-FRAP) were used. Principal component analysis (PCA) was performed to analyze the similarities between the tested honey samples and the relationship between defined statistically significant variables. Using the Kaiser criterion, the two principal components (PCs) accounting for 91.87% of the total variance were chosen: PC1 (including protein content, diastase number, acid phosphatase activity, total phenolic content (TPC), and antioxidant activity (FRAP assay)) and PC2 (electrical conductivity and free acidity), explained 70.19 and 21.68% of the variance, respectively (Figure 2a).

The honey samples were divided into four separate groups (Figure 2b). Samples with the low values of tested parameters are located on the upper-right part of the graph. These were mainly imported samples (linden, multifloral, and acacia) which exhibited lower values of studied parameters compared to samples from ecological apiaries, especially the parameters that are responsible for the health-promoting properties of honey (such as enzymatic and antioxidant activity). A particularly significant difference was observed in the case of buckwheat honey (BC vs. B1 and B2 location on the plot) but linden and multifloral honey are also located in two different sides of the plot (MC and LC left side of PC1, M1, M2, L1, and L2 right side of PC1). Buckwheat honey from ecological apiary was characterized by the highest amount of protein and total phenolic content and diastase number, as well as highest antioxidant and acid phosphatase activity. These variables were highly positively correlated, with a Pearson correlation coefficient *r* above 0.86. Honeydew honey samples are located in the bottom part of the graph, which is linked to PC2. These honey samples had the highest electrical conductivity. This parameter was only correlated with free acidity (*r* = 0.71). This means that this variable depended more on botanical origin than the place where the samples were bought.

Clear separation of imported samples from those bought in ecological apiaries was not observed, because many variables were varietal-dependent. However, if we consider the analysis of individual honey varieties, variables linked to PC1 can be considered as parameters used for the differentiation of samples of low quality.

The stepwise linear discriminant analysis (LDA) was applied to determine which variables could be used to distinguish imported honey samples available on the Polish market from their ecological local counterparts. The best discriminant variables were selected depending on their influence on the classification of samples based on the Wilks′ lambda criterion. Seven significant parameters were used as independent variables, and the geographical origin of the sample was chosen as a dependent variable. The results show that only two variables, protein content and diastase number, were found to be significant (*p* < 0.05) for the discrimination of tested honey samples. One discriminant function was formed: Wilk’s lambda = 0.495, χ^2^= 9.833, df = 2, and *p* = 0.07. The discriminant function was used for the classification of honey samples according to the place of origin because it explained 100% of the total variance, providing an eigenvalue higher than 1.

According to the classification matrix, all imported samples were classified correctly (100% correct classification rate), while for local honeys the classification rate was 80%. One sample (acacia honey) was incorrectly classified (Table 3). The tested acacia honey (AC) was characterized by low enzymatic activity and low protein content, at a very similar level as that found in imported honey. Moreover, in PCA this sample was grouped with imported honey. Based on the obtained results, it can be stated that protein content and diastase number could be considered as markers for the identification of poor-quality samples within the specific honey variety. Furthermore, stepwise linear discriminant analysis proved to be an effective tool in distinguishing imported blends and local organic honeys (100% correct classification). Similarly, stepwise LDA was successfully used by Manzanares et al. [6], who differentiated honeydew from blossom honey based on physicochemical parameters.

## 3. Materials and Methods

### 3.1. Honey

Eleven imported honeys available on the Podkarpackie market in 2018 labeled as mixtures of honeys originating in the EU and not originating in the EU were used. As control samples, five raw local honeys produced in organic apiaries in the Podkarpackie Voivodeship were used. Local honeys were selected as representative samples based on our earlier study. Honey was kept in a dark place at room temperature until analysis. The used markings, varieties, origin, and appearance of tested honeys samples are listed in Table 4.

### 3.2. Refractometric Determination of the Water Content in Honey

The determination of the water content was done by the refractometric method, using a RHN1-ATC refractometer (refraktometr.eu, Hradec Kralove, Czech Republic).

### 3.3. Active and Free Acidity

For the determination of acidity, 20% solutions of honey in distilled water were prepared. To determine active acidity, a pH measurement was used using a CP-401 pH meter (Elmetron, Zabrze, Poland). To determine the free acidity, 50 mL of 20% honey solution was titrated by 0.1 M NaOH to reach a pH of 8.3 measured by pH meter. The results were expressed in mval/kg.

### 3.4. Conductivity

To determine the specific electrical conductivity, 20% solutions of honey in distilled water were used. The conductivity of each honey solution was measured using a conductometer CP-401 (Elmetron, Zabrze, Poland) and the results (in mS/cm) were calculated using a conductivity constant (K = 0.938 cm^−1^).

### 3.5. HMF Determination

The determination of 5-hydroxymethylfurfural (HMF) content was carried out by HPLC in accordance with the guidelines of the regulations of the Polish Ministry of Agriculture and Development of Rural Areas [39]. HPLC analysis was performed at the Plant Biotechnology Laboratory “Aeropolis” using a Gilson chromatograph (Gilson Inc., Middleton, WI, USA) equipped with a binary pump (Gilson 322), DAD detector (Gilson 172), column thermostat (Knauer, Berlin, Germany), and autosampler with a fraction collector (GX-271 Liquid). The separation was carried out using a Knauer Eurosphere II RP-18H 100-5 column (250 × 4.6 mm) with a pre-column (Gilson) at 35 °C, with mobile phase water: methanol (90:10, *v/v*), the isocratic flow was 1 mL/min, analysis time was 15 min, injection volume was 20 μL, and the detection was carried out at wavelength λ = 285 nm. The method was calibrated for the HMF standard (Sigma-Aldrich, Saint Louis, MO, USA) in the range of 0.25 to 6 μg (y = 5123.8x, R^2^ = 0.9989).

### 3.6. Total Protein Content

Protein concentration in the tested honey samples was determined by the Bradford method [40], using 10% solutions of all tested honeys. To 20 μL of honey solution, 1 mL of Bradford′s reagent (BioRad, Hercules, CA, USA) was added and mixed thoroughly. Then, after 5 min the absorbance at λ = 595 nm was measured against a blank using a Biomate 3 spectrophotometer (Thermo Scientific, Waltham, MA, USA). The protein content of the samples was calculated based on the calibration curve (y = 0.0011x, R^2^ = 0.992) made for bovine albumin in the range of 62.5 to 1000 μg.

### 3.7. Diastase Number Determination

Diastase number was determined by a spectrophotometric method with the Phadebas Honey Diastase test (Magle AB, Lund, Sweden) according to the manufacturer′s instructions. Five ml of a 1% honey solution in 0.1 M acetate buffer was heated for 5 min at 40 °C in a water bath. A Phadebas Honey Diastase test tablet was then added to each sample and after thorough mixing, incubated at 40 °C for 30 min. Then, 1 mL 0.5 M NaOH was added, mixed, and filtered into tubes and the absorbance of the filtrate was measured at wavelength λ = 620 nm against a blank (acetate buffer) using a Biomate 3 spectrophotometer (Thermo Scientific, Waltham, MA, USA). The values of the diastase number were calculated from the formulas: Equation (1) when the value of the diastase number did not exceed 8:

DN = 35.2 × A − 0.46(1)

or Equation (2) when diastase number was above 8:

DN = 28.2 × A + 2.64(2)

### 3.8. Acid Phosphatase Activity Assay

Acid phosphatase activity was determined using 4-nitrophenyl phosphate as a substrate, according to Alonso Torre et al. [29] with slight modification. The substrate solution of 5 mM in 0.2 M citrate buffer, pH = 4.5, was used. A total of 100 μL of the test sample (20% *w/v* honey in water) was mixed with 100 μL of a substrate and incubated for 10 min at 37 °C. After this time, 1 mL of 0.25 M NaOH was added to all samples and the absorbance at λ = 400 nm was measured using a Biomate 3 spectrophotometer (Thermo Scientific, Waltham, MA, USA). Acid phosphatase activity was expressed in μmol/g × min, using the molar extinction coefficient 18,000 dm^3^/mol × cm.

### 3.9. Total Phenolic Content Determination

Total phenolic content was determined by the Folin–Ciocalteu method as per Singleton and Rossi [41]. In the test tube, 0.2 mL of 5% honey solution, 1 mL of Folin–Ciocalteu reagent (10%), and 0.8 mL of 7.5% Na_2_CO_3_ were mixed. After 2 h, the absorbance of the test samples against a blank was measured at 760 nm using a Biomate 3 spectrophotometer (Thermo Scientific, Waltham, MA, USA). The total content of polyphenols was expressed as gallic acid equivalents, using a calibration curve made in the concentration range of 0 to 100 mg/dm^3^ (y = 0.0555x, R^2^ = 0.9976).

### 3.10. Antioxidant Assays

For antioxidant potential determination, two methods (DPPH radical scavenging test and FRAP reducing power assay), frequently used in honey analyses, were selected.

#### 3.10.1. DPPH Test

DPPH (2, 2-diphenyl-1-picrylhydrazyl) radical inhibition was measured according to the assay described by Dżugan et al. [7]. A solution of DPPH radical (1.8 mL) was added to the proper samples (0.2 mL of 5% honey solution), and after 30 min absorbance (A) was measured at a wavelength λ = 517 nm relative to the control (A0). The percentage inhibition of (% A) DPPH radical was calculated from the Equation (3):

[% A] = (A0 − A)/A0·100%(3)

#### 3.10.2. FRAP Assay

The FRAP assay (ferric reducing ability of plasma) was carried out as per Bertoncelj et al. [42]. The FRAP reagent contained 2.5 mL of a 10 mM 2,4,6-tripyridyltriazine (TPTZ) solution in 40 mM HCl, 2.5 mL of 20 mM FeCl_3_, and 25 mL of 0.3 M acetate buffer (pH 3.6). In the test tube, 0.2 mL of diluted honey (5% in distilled water) was mixed with 1.8 mL of FRAP reagent. After 10 min of incubation at 37 °C, absorbance at λ = 593 nm was measured against a blank with the use of a Biomate 3 spectrophotometer (Thermo Scientific, Waltham, MA, USA). The results were expressed as μmol of Trolox (TE) equivalents per kilogram of honey (μmol/kg), based on the calibration curve (y = 0.026x, R^2^ = 0.998) prepared for 0.1 mM Trolox in the range of 15 to 200 nmol.

### 3.11. Electrophoretic Analyses

#### 3.11.1. Native Protein Electrophoresis

All the honey samples were diluted with water in a ratio of 1 g of honey per 1 mL of deionized water and mixed thoroughly. A pinch of bromphenol blue was added to the samples as an electrophoretic indicator and 20 μL of the prepared sample was placed in each well. Electrophoresis was carried out on polyacrylamide native gels (10% separating gel and 5% stacking gel, both without SDS) using Tris-glycine running buffer in Mini-Protean II apparatus (Bio-Rad Laboratories, Hercules, CA, USA). The separation was carried out for 2.5 h at 100 V and after electrophoresis, the gels were incubated overnight in a colloidal solution of CBB G-250 [43] and then destained with deionized water for 24 h.

#### 3.11.2. Amylase Electrophoretic Detection

Electrophoresis was performed as described above with one modification—separating gel containing 0.2% of starch was prepared for amylase zymography. Staining for amylases was performed according to Rafiei et al. [44] with slight modifications. When the separation was completed the gels were washed twice with 1% Triton X-100 solution and once with water (10 min each time). Next, gels were washed with 0.25 M acetate buffer, pH 5.5, and placed in a heater (37 °C) overnight. During this time the amylases hydrolyzed the starch present in the gel. The next day, the gels were covered with a solution of iodine in potassium iodide (Lugol′s solution), which resulted in the dark coloration of the whole gel, except the places where the amylases hydrolyzed the starch.

#### 3.11.3. Acid Phosphatase Electrophoretic Detection

Preparation of samples, gels, and electrophoresis was carried out analogously to that for native proteins. Staining for acid phosphatases was performed as per Kalinowski et al. [45]. After the separation, the gels were rinsed twice with 1% Triton X-100 solution for 10 min and with water, also for 10 min. Next, the gels were covered with a solution of 0.5% α-naphthyl phosphate, 0.01% Fast Blue RR and 0.5% polyvinylpyrrolidone, in 0.01 M acetate buffer, pH 5.5, and left overnight. The next day the gels were rinsed with distilled water. Dark bands were observed in places where the migration of proteins with acid phosphatase activity stopped.

### 3.12. Statistical Analysis

Each honey sample was analyzed in triplicate and the results were expressed as means ± standard deviations (SD). The significant differences were obtained by *t*-test (*p* < 0.05). MANOVA was applied to all investigated parameters, as a pre-treatment procedure, to point out the significant data (*p* < 0.05) according to sample origin. Principal component analysis (PCA) and linear discriminant analysis (LDA) using the stepwise method were carried out to differentiate imported honey from raw local honey. The correlation between studied parameters was calculated using Pearson’s correlation test. Statistical analysis was perform using Statistica 13.1 software (StatSoft, Inc., Tulsa, OK, USA).

## 4. Conclusions

Comparing raw local honeys with their imported counterparts (blends) within the same variety, significant differences were found, mainly in antioxidant potential, enzymatic activities, and HMF content, in favor of local honeys. Moreover, Polish honeys were characterized by higher protein content regardless of the variety, excluding acacia honey, which was also confirmed using the native PAGE method. For the first time, the possibility of zymographic identification of native amylase and acid phosphatase isoenzymes in honey was proposed. The usefulness of in-depth protein analysis to assess the quality of honey and to distinguish local and imported honeys was confirmed by multivariate statistical analysis (PCA and LDA). Considering the antioxidant properties and enzymatic activity of honey, responsible for its pro-health value which can be reduced during thermal processing, it can be concluded that raw local honeys are more valuable products than imported honey blends.

## Figures and Tables

**Table 1:** The physicochemical parameters of imported honey blends and raw local honey compared to applicable EU regulations.

Honey Sample		Moisture Content [%]	pH	Free Acidity [mval/kg]	Electrical Conductivity [mS/cm]	HMF Content [mg/kg]
Nectar Honey	A1	17.20 ± 0.00	3.99 ± 0.02 *	16.00 ± 1.40 *	0.40 ± 0.00 *	5.82 ± 0.00 *
	A2	18.75 ± 0.05 *	4.23 ± 0.01 *	10.50 ± 0.70 *	0.31 ± 0.02	35.20 ± 0.00 *
	**AC**	**17.17 ± 0.30**	**3.77 ± 0.03**	**20.85 ± 6.50**	**0.31 ± 0.18**	**22.66 ± 0.00**
	B1	19.80 ± 0.00	4.01 ± 0.02 *	21.50 ± 0.70 *	0.54 ± 0.00 *	6.40 ± 0.60 *
	B2	19.70 ± 0.00	3.83 ± 0.00	23.50 ± 0.70 *	0.54 ± 0.01 *	34.18 ± 0.00 *
	**BC**	**20.50 ± 0.00**	4.04 ± 0.00 *	**26.50 ± 0.00**	**0.43 ± 0.01**	**20.02 ± 0.00**
	L1	20.50 ± 0.10	4.32 ± 0.00	14.50 ± 0.70 *	0.25 ± 0.02 *	19.33 ± 0.00 *
	L2	19.05 ± 0.15 *	4.42 ± 0.02	14.50 ± 0.70 *	0.23 ± 0.02 *	23.23 ± 0.00 *
	**LC**	**20.30 ± 0.10**	**4.13 ± 0.00**	**25.50 ± 0.00**	**0.64 ± 0.00**	**13.20 ± 0.00**
	M1	18.85 ± 0.05 *	4.30 ± 0.02	10.50 ± 2.10 *	0.79 ± 0.02 *	73.92 ± 0.00 *
	M2	18.35 ± 0.05 *	3.75 ± 0.02 *	16.50 ± 0.70 *	0.75 ± 0.01 *	47.55 ± 0.48 *
	**MC**	**20.20 ± 0.00**	**4.11 ± 0.00**	**29.50 ± 0.00**	**0.67 ± 0.00**	**12.50 ± 0.00**
	Applicable limits [2]	20%		<50	<0.8	<40
Honeydew Honey	H1	17.55 ± 0.05 *	4.54 ± 0.02 *	29.50 ± 0.70 *	1.70 ± 0.01 *	11.78 ± 0.12 *
	H2	17.55 ± 0.05 *	4.44 ± 0.01 *	38.00 ± 1.40 *	1.95 ± 0.06 *	35.38 ± 0.36 *
	H3	18.75 ± 0.15 *	4.68 ± 0.00 *	29.50 ± 2.10 *	1.99 ± 0.07 *	15.04 ± 0.14
	**HC**	**19.90 ± 0.00**	**3.64 ± 0.00**	**30.50 ± 0.00**	**1.16** **± 0.00**	**19.80** **± 0.00**
	Applicable limits [2]	20%		<50	>0.8	<40

* Means marked with the symbol differ statistically significantly from a suitable high-quality local control sample (marked with bold): AC for acacia, BC for buckwheat, LC for linden, MC for multifloral, and HC for honeydew honey (*t*-test, *p* < 0.05).

**Table 2:** The content of soluble protein, enzyme activity, and antioxidant activity of imported honey blends and raw local honey.

Honey Sample	Protein Content [mg/100 g]	Enzymatic Activity		Antioxidant Activity		
		Diastase Activity (as Diastase Number DN)	Acid Phosphatase Activity (mmol/g/min)	Total Phenolic Content (mg GAE/kg)	FRAP (μmol TE/kg)	DPPH (% Radical Inhibition)
A1	27.27 ± 2.57 *	8.3 ± 2.3 *	5.0 ± 1.9	58.56 ± 0.56 *	203.85 ± 2.04 *	10.82 ± 0.22 *
A2	19.09 ± 2.57 *	2.7 ± 0.6 *	7.5 ± 2.6	98.20 ± 2.95 *	317.31 ± 6.35 *	11.51 ± 0.12 *
**AC**	**37.73 ± 0.64**	**5.63 ± 1.8**	**7.7 ± 0.5**	**140.81 ± 11.41**	**531.96 ± 18.50**	**19.64 ± 6.13**
B1	224.55 ± 6.43 *	13.9 ± 3.8 *	20.7 ± 0.4 *	516.22 ± 5.02 *	1113.46 ± 33.40 *	33.99 ± 1.02 *
B2	175.46 ± 14.14 *	10.3 ± 2.2 *	19.5 ± 1.7 *	653.15 ± 6.53 *	623.08 ± 11.98 *	39.55 ± 4.00 *
**BC**	**475.456 ± 3.86**	**66.43 ± 5.7**	**31.8 ± 2.6**	**2075.70 ± 19.90**	**4973.10 ± 46.73**	**59.69 ± 2.51**
L1	23.18 ± 0.64 *	4.3 ± 1.1 *	6.7 ± 0.9 *	125.23 ± 1.50 *	615.38 ± 12.31 *	17.76 ± 1.60 *
L2	23.634 ± 3.856 *	8.0 ± 1.2 *	5.2 ± 1.2 *	102.70 ± 1.13 *	607.69 ± 18.23 *	17.92 ± 0.36 *
**LC**	**89.55 ± 1.93**	**28.94 ± 3.8**	**17.8 ± 2.6**	**436.90 ± 4.25**	**1555.80 ± 14.82**	**23.30 ± 0.76**
M1	10.91 ± 0.00 *	2.7 ± 0.7 *	9.8 ± 1.7 *	161.26 ± 2.01 *	425.00 ± 8.50 *	13.73 ± 0.14 *
M2	53.18 ± 3.21 *	3.1 ± 0.9 *	10.3 ± 1.4	117.20 ± 1.18 *	334.62 ± 6.69 *	13.41 ± 0.11 *
**MC**	**133.18 ± 4.50**	**29.51 ± 4.0**	**11.5 ± 0.2**	**496.80 ± 5.00**	**1470.20 ± 14.93**	**19.52 ± 0.26**
H1	89.09 ± 6.43 *	16.3 ± 2.2 *	16.7 ± 0.5	310.81 ± 2.89 *	1390.38 ± 41.71 *	79.37 ± 4.76
H2	105.456 ± 21.86 *	12.5 ± 1.9 *	17.3 ± 2.8 *	597.30 ± 11.95	1934.62 ± 77.38 *	72.32 ± 0,71
H3	70.46 ± 8.36	14.6 ± 1.0	16.3 ± 1.9	568.47 ± 6.25 *	1488.46 ± 29.77 *	12.73 ± 0.10 *
**HC**	**70.91 ± 2.57**	**17.45** **± 2.9**	**14.0 ± 1.9**	**646.80 ± 5.87**	**1526.00 ± 16.97**	**69.58 ± 0.35**

* Means marked with the symbol differ statistically significantly from a high quality local control sample (marked with bold): AC for acacia, BC for buckwheat, LC for linden, MC for multifloral, and HC for honeydew honey (*t*-test, *p* < 0.05).

**Table 3:** Results of stepwise discriminant analysis (LDA) for all the samples considered of different origin, and classification matrix for individual honey samples.

Original Group	Predicted Classification (Number of Samples)		Correct Classification (%)
	Imported	Local	
imported	11	0	100
local	1	4	80
Total	12	4	93.75
Honey Samples	Original Group		Predicted Classification
A1	imported		imported
A2	imported		imported
AC *	local		imported
B1	imported		imported
B2	imported		imported
BC	local		local
L1	imported		imported
L2	imported		imported
LC	local		local
M1	imported		imported
M2	imported		imported
MC	local		local
H1	imported		imported
H2	imported		imported
H3	imported		imported
HC	local		local

* Incorrect classifications are marked.

**Table 4:** List of honeys used for research.

Symbol	Variety	Type	Origin	Color	Crystallization State
A1	acacia	blend, imported	from outside the EU	white	liquid
A2	acacia	blend, imported	EU and from outside the EU	white	liquid
AC	acacia	raw, local	Podkarpackie, Poland	white	liquid
B1	buckwheat	blend, imported	EU and from outside the EU	dark amber	partially crystallized
B2	buckwheat	blend, imported	EU and from outside the EU	dark amber	crystallized
BC	buckwheat	raw, local	Podkarpackie, Poland	dark amber	partially crystallized
L1	linden	blend, imported	EU and from outside the EU	white	half crystallized
L2	linden	blend, imported	EU and from outside the EU	extra light amber	half crystallized
LC	linden	raw, local	Podkarpackie, Poland	extra light amber	liquid
M1	multifloral	blend, imported	EU and from outside the EU	amber	crystallized
M2	multifloral	blend, imported	EU and from outside the EU	light amber	crystallized
MC	multifloral	raw, local	Podkarpackie, Poland	light amber	crystallized
H1	honeydew	blend, imported	EU and from outside the EU	dark amber	crystallized
H2	honeydew	blend imported	EU	dark amber	liquid
H3	honeydew	blend, imported	EU	dark amber	liquid
HC	honeydew	raw, local	Podkarpackie, Poland	dark amber	crystallized

## Data Availability

The data presented in this study are available in the article.

## Sample Availability

Samples of honeys are available from the authors for a limited time.
